# The Influence of Interleukin 6 Knockout on Age-Related Degenerative Changes in the Cerebellar Cortex of Mice

**DOI:** 10.3390/cells14070532

**Published:** 2025-04-02

**Authors:** Magdalena Wiktoria Cieślińska, Izabela Bialuk, Magdalena Dziemidowicz, Beata Szynaka, Joanna Reszeć-Giełażyn, Maria Małgorzata Winnicka, Tomasz Andrzej Bonda

**Affiliations:** 1 Department of General and Experimental Pathology, Medical University of Białystok, Mickiewicza 2c, 15-222 Białystok, Poland; 2 Department of Histology and Embryology, Medical University of Bialystok, Waszyngtona 13, 15-269 Białystok, Poland; 3 Department of Medical Pathomorphology, Medical University of Bialystok, Waszyngtona 13, 15-269 Białystok, Poland

**Keywords:** IL-6 deficiency, Purkinje cells, aging, p53

## Abstract

This study investigates age-related neurodegeneration in the cerebellar cortex, emphasizing the role of IL-6 deficiency in preserving Purkinje cells. We found that apoptosis plays a minimal role in Purkinje cell loss by using 4-month- and 24-month-old wild-type (WT) and IL-6 knockout (IL-6KO) mice. At 24 months, WT mice exhibited severe Purkinje cell degeneration, including atrophic cell bodies, eosinophilic cytoplasm, pyknotic nuclei, mitochondrial disruption, and increased levels of lipofuscin-rich lysosomes. In contrast, IL-6KO mice showed fewer lysosomes, reduced mitochondrial damage, and less neuronal atrophy, indicating a neuroprotective effect. Lower p53 expression and decreased levels of its downstream effectors (p21, and Bax) in IL-6KO mice correlated with reduced cellular stress. Minimal changes in apoptotic markers (Bax and caspase-3) further reinforce the limited role of apoptosis. Neuroinflammation, marked by elevated GFAP, was prominent in aged WT mice but attenuated in IL-6KO mice. Reduced p53 accumulation, less severe neuroinflammation, and preserved metabolic homeostasis in IL-6KO mice correlated with improved Purkinje cell survival. These findings suggest that IL-6 accelerates neurodegeneration via p53-associated stress and inflammation, while IL-6 deficiency mitigates these effects. Targeting IL-6 signaling through anti-inflammatory strategies or IL-6 inhibition may offer a therapeutic approach for age-related neurodegenerative disorders.

## 1. Introduction

The aging process is an inevitable phenomenon, the basis of which is embedded in the life of every multicellular organism, including humans. Age-related changes in the central nervous system (CNS) result in impaired motor function and progressive cognitive decline. Cellular, subcellular, and molecular changes, such as altered gene expression patterns, precede the clinical manifestations of aging. Therefore, research on disorders that anticipate the appearance of age-related clinical manifestations can provide the basis for attempts to alleviate or delay the onset of aging.

Interleukin 6 (IL-6) is an important CNS cytokine influencing physiological and pathological processes. It regulates synaptic and cognitive functions, supports neuronal development, and plays a significant role in neuroimmune responses. In the CNS, the primary sources of IL-6 are astrocytes and microglia. However, some neurons have also been shown to produce IL-6 [1,2]. IL-6 exerts biological effects through a receptor system consisting of the specific IL-6 receptor (IL-6R) and the membrane-bound gp130 protein, also known as the IL-6 signal transducer (IL-6ST). In an alternative signaling mechanism, a soluble form of IL-6R (sIL-6R) can bind to gp130, triggering intracellular signal transduction and activating pathways such as JAK-STAT, MAP kinase, or PI3K. While the expression of IL-6R is limited to specific cell populations, gp130 is expressed ubiquitously. Therefore, some neurons are unresponsive to IL-6 alone but can be stimulated by sIL-6R [2].

In the cerebellar cortex, Purkinje cells, granular cells, inhibitory interneurons, and glial cells express both the IL-6 receptor and gp130 protein, indicating that IL-6 plays an important role in cerebellar development and function [3,4]. The expression of IL-6 by Purkinje cells and adjacent Bergmann glial cells suggests an autocrine and paracrine mode of action, highlighting the close functional relationship between these cells [5,6]. Under normal conditions, IL-6 levels in the CNS are low. However, transgenic animals overexpressing IL-6 in astrocytes (GFAP IL-6) exhibit significant cerebellar abnormalities, including Purkinje cell loss, cerebellar atrophy, astrocytosis, and inflammatory cell accumulation within cerebellar grooves [7]. These mice also show neurological symptoms, suggesting impaired cerebellar-dependent motor control. Studies conducted by Nelson et al. [4] demonstrated that the direct action of IL-6 on Purkinje cells caused electrophysiological disturbances in these mice. These authors suggest that disrupted Purkinje cell physiology in IL-6-overexpressing mice might be caused by the altered expression of proteins involved in spontaneous electrical activity, including ion channel proteins, regulatory proteins, and intracellular signal transduction pathways [8,9]. In addition, chronic exposure of cerebellar cortex cells to IL-6 has been shown to cause disturbances in calcium homeostasis and changes in electrical activity [10].

The expression of IL-6 increases with age and in neurodegenerative diseases, accelerating the aging process [11]. Age-related changes in the central nervous system (CNS) are associated with neuronal loss [12], with the cerebellum containing approximately 80% of CNS neurons in primates [13]. Among the most vulnerable neurons are Purkinje cells, whose degeneration is linked to cerebellar dysfunction. Up to 30% of Purkinje cells may be lost during aging, contributing to postural instability [14], impaired motor function [15,16,17], and cognitive decline [13,17,18].

IL-6 plays a key role in neurodegenerative diseases such as Alzheimer’s disease (AD) [18] and Parkinson’s disease (PD) [19]. In AD, IL-6 promotes neuroinflammation, amyloid-beta aggregation, and tau protein hyperphosphorylation, all of which contribute to neuronal damage [18]. In PD, elevated IL-6 levels in the nigrostriatal region and cerebrospinal fluid suggest its involvement in disease progression. Reactive astrocytes release IL-6, which can induce dopaminergic neuron toxicity via IL-6R activation and the STAT3 pathway [19]. As regulators of motor coordination and cognitive function, Purkinje cells are implicated in various neurological disorders, including ataxia, epilepsy, and cognitive impairments. Recent studies on IL-6 and cerebellar aging suggest that constitutive IL-6 production leads to a fivefold increase in cerebellar microglia. Aging alone results in reduced cerebellar volume and a 50% decline in Purkinje cells, with chronic neuroinflammation further exacerbating this loss [20].

Our study aims to determine whether IL-6 deficiency influences the apoptotic death of cerebellar cortical cells by examining the molecular mechanisms regulating apoptosis in young adult and aged mice. Given that degenerating cells are primarily eliminated through apoptosis, we investigate the role of IL-6 in neuroinflammation and age-related neurodegeneration, focusing on key molecular markers associated with gliosis, apoptosis, and neuronal survival. IL-6 is a pro-inflammatory cytokine implicated in CNS injury and neurodegenerative processes, and its absence (IL-6KO) may confer neuroprotection by reducing astrocyte reactivity, as indicated by lower GFAP expression [21]. Additionally, IL-6-driven oxidative stress and inflammation may influence p53 activation, a key regulator of cellular stress responses. By assessing p53 and its downstream targets, including MDM-2, p21, Bax, and Bcl-2, we aim to determine whether IL-6KO shifts the cellular environment towards enhanced neuronal death or survival [22,23,24]. NSE, a marker of neuronal integrity, will give its relevance to metabolic activity and neurodegeneration [22,23,24,25]. By evaluating these molecular pathways, our study seeks to clarify the impact of IL-6 deficiency on neuronal health and provide insights into its potential as a therapeutic target for neurodegenerative conditions.

## 2. Materials and Methods

### 2.1. Animals

Naïve, male 4- and 24-month-old C57BL/6J IL6^-/-tm1Kopf^ (IL-6KO) and C57BL/6J (WT) mice were used. Animals were obtained from the Center of Experimental Medicine of the Medical University of Bialystok and were originally purchased from Jackson Laboratories (Bar Harbor, ME, USA). Each group consisted of six animals. All procedures were performed under Directive 2010/63/EU of the European Parliament and approved by the Local Animal Ethics Committee. Mice were housed under controlled conditions: temperature (22 °C ± 1 °C) and lighting (12:12 dark–light cycle), with constant access to standard chow and water. Mice were sacrificed by cervical dislocation, and brains were immediately excised. Tissues were fixed in phosphate-buffered formalin for paraffin embedding or frozen in liquid nitrogen for Western blot analysis. Genotypes were confirmed by PCR, as previously described [26].

### 2.2. Histology

Paraffin-embedded tissues were sectioned into 5 μm slices. Standard hematoxylin and eosin (H+E) staining was performed for histological assessment. Additional immunofluorescence and immunohistochemistry analyses were conducted, as described below. To reduce bias, researchers performing the analyses of images were blinded to the experimental conditions.

### 2.3. Immunofluorescence

The sections were subjected to deparaffinization, rehydration, and epitope retrieval using proteinase K (1:800 solution) and blocking with 10% donkey serum in phosphate-buffered saline (PBS, pH 7.4) for 1 h at room temperature (RT). The primary antibody against GFAP (ab7260, Abcam, Cambridge, UK) was applied at 1:500 in PBS for 90 min. at RT. Next, the sections were washed and incubated with a biotin-conjugated secondary antibody (donkey anti-rabbit IgG, 711-065-152, Jackson Immuno Research Laboratories, West Grove, PA, USA) at a 1:200 dilution in PBS for 1 h at RT, followed by washing with PBS with 0.1% Tween 20. Subsequently, the sections were incubated with streptavidin-Alexa Fluor^®^ 488 (S32354, Life Technologies, Carlsbad, CA, USA) at a dilution of 1:1000 in PBS for 40 min. at RT, washed, and counterstained with HOECHST 33258 (Sigma-Aldrich) in PBS for 2 min at RT. Slides were coverslipped in Dako Mounting Medium and evaluated using an Olympus BX41 microscope equipped with the epifluorescence module, using UPlanFLN 40×/0.75 and Olympus PlanCN 20×/0.40 objectives. The microphotographs were taken using Olympus XC30 CCD camera and Cell^D software (Olympus, Tokio, Japan).

### 2.4. Immunohistochemistry

Epitope retrieval was carried out in the PT Link at a high pH (pH = 9.0) after sections of deparaffinization and rehydration. Next, slides were stained in the Autostainer Link system (DAKO, Kraków, Poland). Polyclonal antibody against the active form of caspase-3 protein (Anti-Caspase-3 antibody (E-8), sc-7272, Santa Cruz Biotechnology Dallas, TX, USA) in 1:100 dilution was used. Visualization reagent FLEX/EnVision (DAKO) was applied for 30 min. and followed by DAB (DAKO). Slides were assessed using bright-field microscopy with an Olympus BX41 microscope.

### 2.5. TUNEL Method

After deparaffinization, the sections were subjected to fluorescein terminal deoxynucleotidyl transferase-mediated dUTP-marker nick-end labeling (TUNEL) using ApopTag^®^ Fluorescein In Situ Apoptosis Detection Kit (S7110,Millipore, Burlington, MA, USA), according to the manufacturer’s protocol. Counterstaining of cell nuclei was performed using HOECHST 33258 (Sigma, St. Louis, MO, USA). All slides were analyzed and photographed in a fluorescence microscope (Olympus BX 41).

### 2.6. Transmission Electron Microscopy (TEM)

Approximately 1 mm^3^ of the cerebellar cortex was taken from 3 animals from both young adult and aged groups. Samples were fixed immediately in a mixture of 2.5% glutaraldehyde and 2% paraformaldehyde in 0.1 M cacodylate buffer (CB). Next, samples were washed in CB at 4 °C and postfixed in 1% osmium tetroxide in CB for 1 h at 4 °C. In the next step, samples were dehydrated through a graded series of ethanol and embedded in Glycid ether 100 (Serva, Heidelberg, Germany). Ultrathin sections were contrasted with uranyl acetate and lead citrate and mounted on nickel grids. For the evaluation of electronograms, a transmission electron microscope OPTON 900 PC (Zeiss, Oberkochen, Germany) was used. Electronograms were acquired and analyzed using the ImageSP software (Zeiss, Germany).

### 2.7. Western Blot

The cerebellar cortex was homogenized on ice in RIPA buffer (Sigma) containing protease and phosphatase inhibitors (Sigma) using a manual homogenizer. The homogenates were centrifuged at 4 °C for 10 min at 8000 rpm. The protein concentration in the supernatant was measured using the Bradford method (Bio-Rad, Hercules, CA, USA). An amount of 40 μg of the protein sample was subjected to SDS-PAGE, according to the method of Laemmli, in 15% polyacrylamide gel and blotted onto nitrocellulose membranes 0.2 mm (BioRad). Equal loading was confirmed using Ponceau Red staining (Sigma). Membranes were blocked with 5% BSA (Sigma) or 5% non-fat dry milk for 1 h at RT. Primary antibodies recognizing mouse’s GFAP (Abcam, ab7260, 1:10,000), p53 (BD, 554166, 1:2000), MDM-2 (Thermo Scientific, PA5-27209, 1:2000), Bax (Cell Signaling, Danvers, MA, USA, 2772S, 1:500), Bcl-2 (Cell Signaling, 2870S, 1:1000), p21 (Thermo Scientific, Waltham, MA, USA p21/Cip, PA1-30399, 1:1000), NSE (Abcam, ab 53025, 1:10,000), and α-Tubulin (B-7, Santa Cruz Biotechnology, sc-5286, 1:8000) were used. Secondary antibodies were conjugated with horseradish peroxidase (anti-rabbit IgG-HRP, AbD Serotec—STAR54 and anti-mouse IgG-HRP, Sigma-A9303). Blots were visualized using enhanced chemiluminescence reaction (Thermo Scientific) and exposed to the X-ray film (X-Omat Blue, Kodak). Films were scanned and quantified using the ImageJ Software v. 1.53a (National Institutes of Health, Bethesda, MD, USA). The results of particular experiments were related to the expression of proteins in the control group, which was set as 100%.

### 2.8. Statistics

Statistical analyses were performed using Statistica 13.0 and GraphPad Prism 5 programs. All data were first assessed for normality using the Shapiro–Wilk test. In the present study, some variables measured did not have a normal distribution. Therefore, some data were analyzed by one-way analysis of variance (ANOVA) with Bonferroni or Tukey’s post hoc tests or by Kruskal–Wallis followed with Dunn’s multiple comparison post hoc tests, when appropriate. Differences were considered significant at *p* < 0.05. *F* values for ANOVA and H values for the Kruskal–Wallis test, degrees of freedom, and *p* values were given only for significant differences. Results are presented as mean value ± standard error of the mean (SEM).

## 3. Results

### 3.1. Morphology of the Mouse Cerebellar Cortex

**Figure 1:**
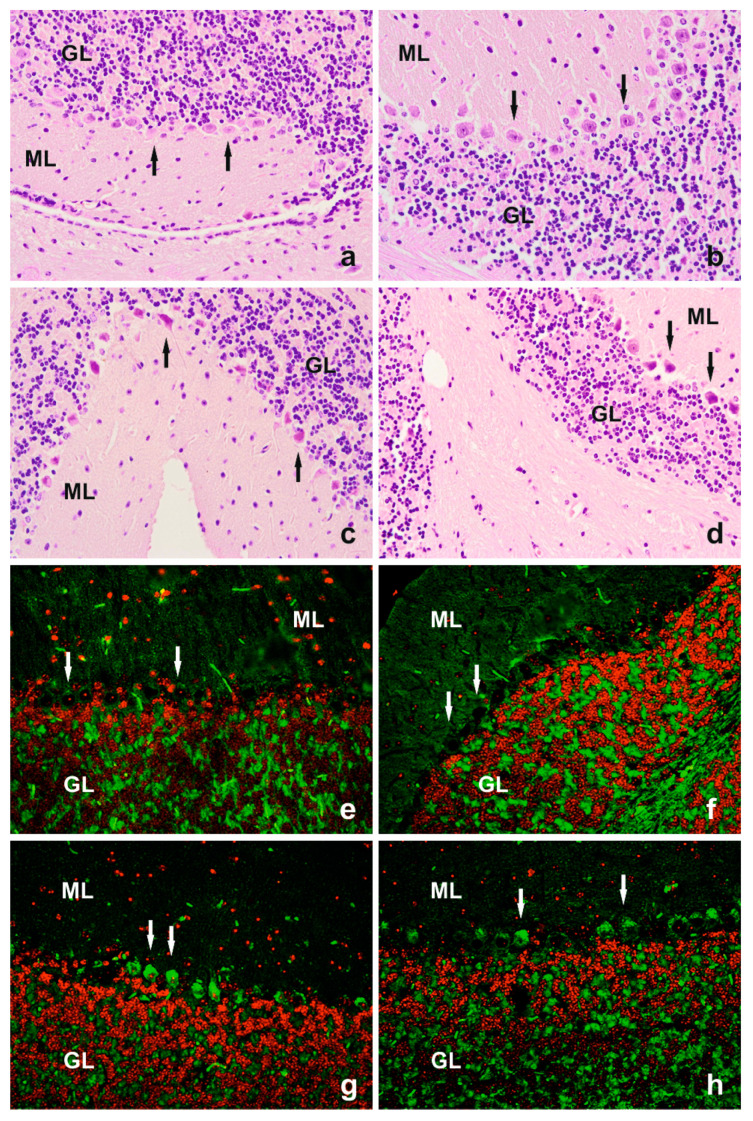
Hematoxylin and eosin staining of mouse cerebellar cortex. (**a**) The 4-month-old WT, (**b**) 4-month-old IL-6KO, (**c**) 24-month-old WT, and (**d**) 24-month-old IL-6KO (400× magnification). Arrows indicate proper Purkinje cells of the middle layer (**a**,**b**) and cells with degenerative changes (**c**,**d**). Autofluorescence of granules in Purkinje cells in the cerebellar cortex of (**e**) 4-month-old WT, (**f**) 4-month-old IL-6KO, (**g**) 24-month-old WT, and (**h**) 24-month-old IL-6KO mice (200× magnification). In perikaryons of Purkinje cells and accompanying Bergmann glial cells in aged animals of both genotypes, autofluorescence of lipofuscin granules was observed. Arrows indicate the Purkinje cells layer. The green color indicates the autofluorescence of glow granules in the cytoplasm of Purkinje cells. The nuclei are shown in red. ML—molecular layer and GL—granular layer.

Hematoxylin and eosin (H+E) staining showed normal cerebellar morphology in the 4-month-old animals of both genotypes (Figure 1a,b). The cerebellar cortex exhibited three well-defined layers with intact cellular content and architecture. The granular layer had tightly arranged cells with spherical, hematoxylin-stained nuclei. The molecular layer displayed loosely arranged cells with visible nuclei and dark nucleoli. The Purkinje cells were organized in a single layer between these layers, characterized by large, flask-shaped bodies containing spherical nuclei with diffuse chromatin and prominent nucleoli. The first portion of the dendritic projections towards the molecular layer was visible in some of these cells.

In the cerebellar cortex of the older animals of both genotypes, no differences were observed in the morphological characteristics of the granular and molecular layers compared to those of the young mice; however, degeneration and loss of Purkinje cells were evident in the 24-month-old animals. In many Purkinje cells, shrinkage of the perikaryon and loss of the characteristic pear-like shape were evident. The cytoplasm in these cells was more eosinophilic, and some of these cells contained pyknotic nuclei. The cross-sections of the cerebellar cortex of the aged animals revealed a local loss of cells within the Purkinje layer. The degenerative changes and the loss of Purkinje cells were less pronounced in the 24-month-old IL-6KO mice than in the age-matched WT animals (Figure 1c,d). Moreover, in the perikaryon of Purkinje cells and the adjacent Bergmann glial cells in the aged animals of both genotypes, autofluorescence of lipofuscin granules was observed (Figure 1g,h).

### 3.2. Transmission Electron Microscopy (TEM)

**Figure 2:**
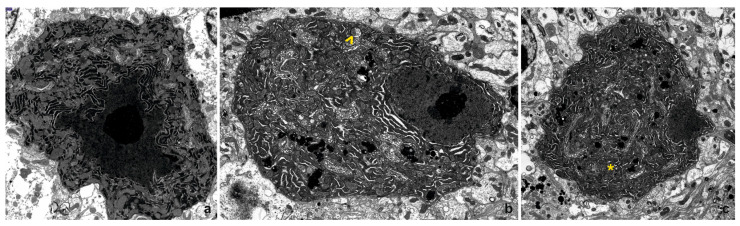
Representative electron photomicrograph of neurons in the cerebellar cortex: (**a**) From a 4-month-old WT mouse. Note the irregular shape of the cell nucleus, with dense chromatin and a large, prominent nucleolus (magnification × 7000). (**b**) From a 24-month-old WT mouse. The cell shows extensive Golgi apparatus cisternae, numerous lysosomes, and lysosomes containing lipofuscin. A mitochondrion surrounded by a membrane and isolated from the cytoplasm is also visible (indicated by ^) (magnification × 4400). (**c**) From a 24-month-old IL-6KO mouse. A cell with a nucleus exhibiting uneven outlines and regularly dispersed chromatin. Mitochondria of various shapes are visible, with some showing a blurred internal structure (indicated by *) (magnification × 3000).

In the 4-month-old WT mice, Purkinje cells exhibit irregular shapes that vary depending on the cross-section, with some displaying a characteristic pear-shaped appearance. The nuclei contain dense chromatin clumps and a prominent nucleolus, while the nuclear membrane frequently forms invaginations, some of which are deep. Mitochondria are typically large, oval, or rod-shaped, with a normal structure characterized by a moderately electron-dense matrix. The Golgi apparatus is well-developed and distributed throughout the cytoplasm, occasionally accompanied by multivesicular bodies. Numerous elongated channels in the rough endoplasmic reticulum (RER) and scattered ribosomes contribute to the cytoplasm’s high electron density (Figure 2a). The cells in the young IL-6KO mice share similar ultrastructural features with those of the young WT mice.

In the 24-month-old WT mice, Purkinje cells show irregular outlines and nuclei that are irregularly shaped, with fewer invaginations compared to younger cells. Chromatin appears more dispersed, while the nucleoli remain large. Mitochondria exhibit varied shapes, some containing slightly translucent matrices or blurred crista structures. Occasionally, individual mitochondria are enclosed by membranes resembling mitophagosomes. The Golgi apparatus is extensive and frequently associated with numerous lysosomes, some containing lipofuscin granules identifiable by their high electron density and distinctive substructure. The cytoplasm remains rich in RER channels and scattered free ribosomes (Figure 2b). In the aged IL-6KO mice, the ultrastructure of Purkinje cells largely resembles that of the aged WT mice. Slightly brightened mitochondria with occasional blurred cristae are present, similar to those observed in aged WT mice. However, fewer lysosomes are observed near the Golgi apparatus compared to what is observed in the aged WT mice (Figure 2c). Despite these differences, the overall cellular architecture remains comparable between the two genotypes in the aged animals.

### 3.3. Expression of Glial Cell Response Marker GFAP

**Figure 3:**
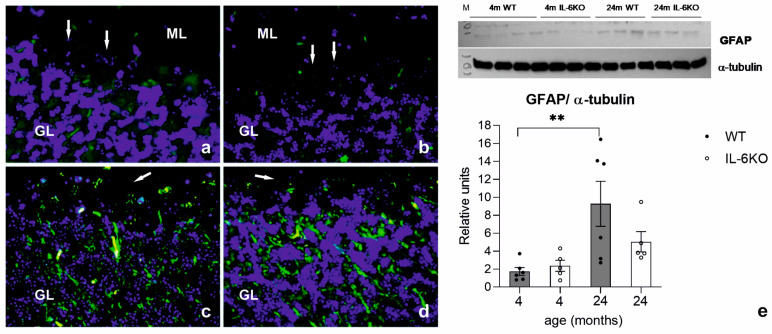
Tissue localization of glial fibrillary acidic protein (GFAP) in cerebellar cortex of (**a**) 4-month-old WT, (**b**) 4-month-old IL-6KO, (**c**) 24-month-old WT, and (**d**) 24-month-old IL-6KO mice evaluated by immunofluorescence (400× magnification). In young adult animals of both genotypes, the immunofluorescence of GFAP observed in cell bodies of astrocytes and their processes was very weak. In contrast, it was much stronger in the cerebellar cortex of 24-month-old animals, especially in WT animals. Arrows indicate the Purkinje cells layer. GFAP is shown in green and the nuclei are shown in blue. ML—molecular layer and GL—granular layer. (**e**) Western blots and densitometric analysis presenting the relative expression of the glial fibrillary acidic protein (GFAP) in cells of the cerebellar cortex of 4- and 24-month-old IL-6-deficient (IL-6KO) and control group (WT) mice. Columns represent the mean ± SEM obtained from 5–6 animals. The amount of GFAP was comparable in both young adult groups and significantly higher in the aged WT group vs. younger WT animals (** *p* < 0.05, ANOVA and Bonferroni post hoc test). Above the graph is a representative immunoblot for GFAP protein shown together with α-tubulin as a loading control. M—marker.

Immunofluorescence revealed weak glial fibrillary acidic protein (GFAP) staining in the astrocytes of the 4-month-old mice from both genotypes (Figure 3a,b). In the aged animals, GFAP expression increased significantly, particularly in the WT mice (Figure 3c,d). Western blot analysis confirmed these findings. While GFAP protein levels in the 4-month-old mice of both genotypes were comparable, an overall increasing trend was observed in the aged mice. This increase was more pronounced in the WT animals, where the ANOVA showed a significant effect (*F*_(3,18)_ = 5.410, *p* = 0.0079). Bonferroni’s post hoc test revealed a significant difference only between the young and aged WT groups (*p* < 0.05), but no difference between aged groups of both genotypes (Figure 3e).

### 3.4. Apoptosis in Mouse Cerebellar Cortex

**Figure 4:**
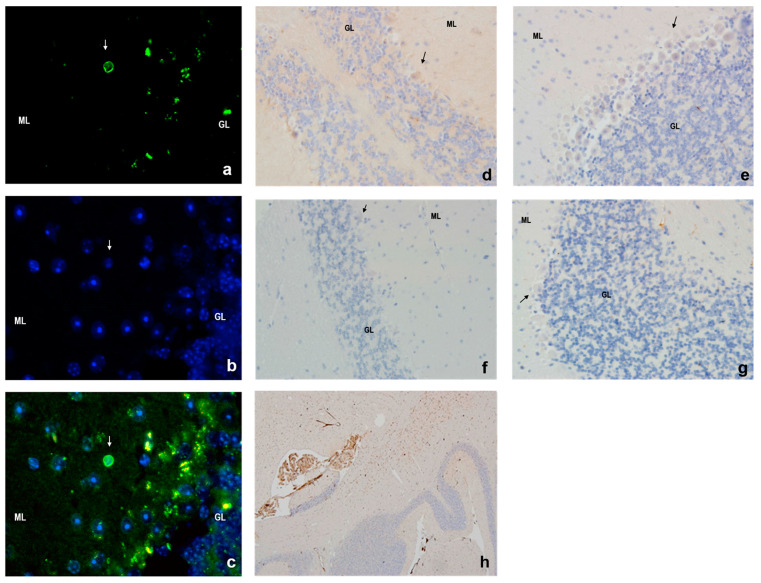
The apoptotic cell death was evaluated by the TUNEL method (**a**–**c**). (**a**) Fluorescent labeling of the free ends 3′ results in DNA degradation in the apoptotic process. (**b**) Fluorescent staining of nuclei. (**c**) Merged image produced from the superimposition of photographs a and b (400× magnification). Arrows indicate the cell during the process of apoptosis. Low apoptotic activity of cerebellar cortex of (**d**) 4-month-old WT, (**e**) 4-month-old IL-6KO, (**f**) 24-month-old WT, and (**g**) 24-month-old IL-6KO mice was in accordance with lack of caspase-3 activity in neurons of all cerebellar layers evaluated by immunohistochemistry (200× magnification). Arrows indicate Purkinje cells. (**h**) Positive control for the presence of immunohistochemical staining of active caspase-3. The Figure shows the image of a positive reaction to the active caspase-3, which is evident in the cerebellar choroid’s plexus, and the lack of a similar reaction in the cells of the cerebellar cortex (40× magnification). ML—molecular layer and GL—granular layer.

The TUNEL assay revealed no apoptotic cells in the cerebellar cortex of the 4-month-old animals and only a few TUNEL-positive cells in the molecular layer of the 24-month-old animals (Figure 4a–c). No TUNEL-positive cells were observed in Purkinje cells. Similarly, active caspase-3 staining was absent in cerebellar neurons across all groups, but it was detected in vascular endothelial cells and cells of the vascular plexus. These findings were consistent with the immunohistochemistry results, which showed no caspase-3 activity in neurons across all cerebellar layers (Figure 4d–h).

### 3.5. Expression of p53, MDM-2, Bax, Bcl-2, p21, and NSE in Mouse Cerebellar Cortex

**Figure 5:**
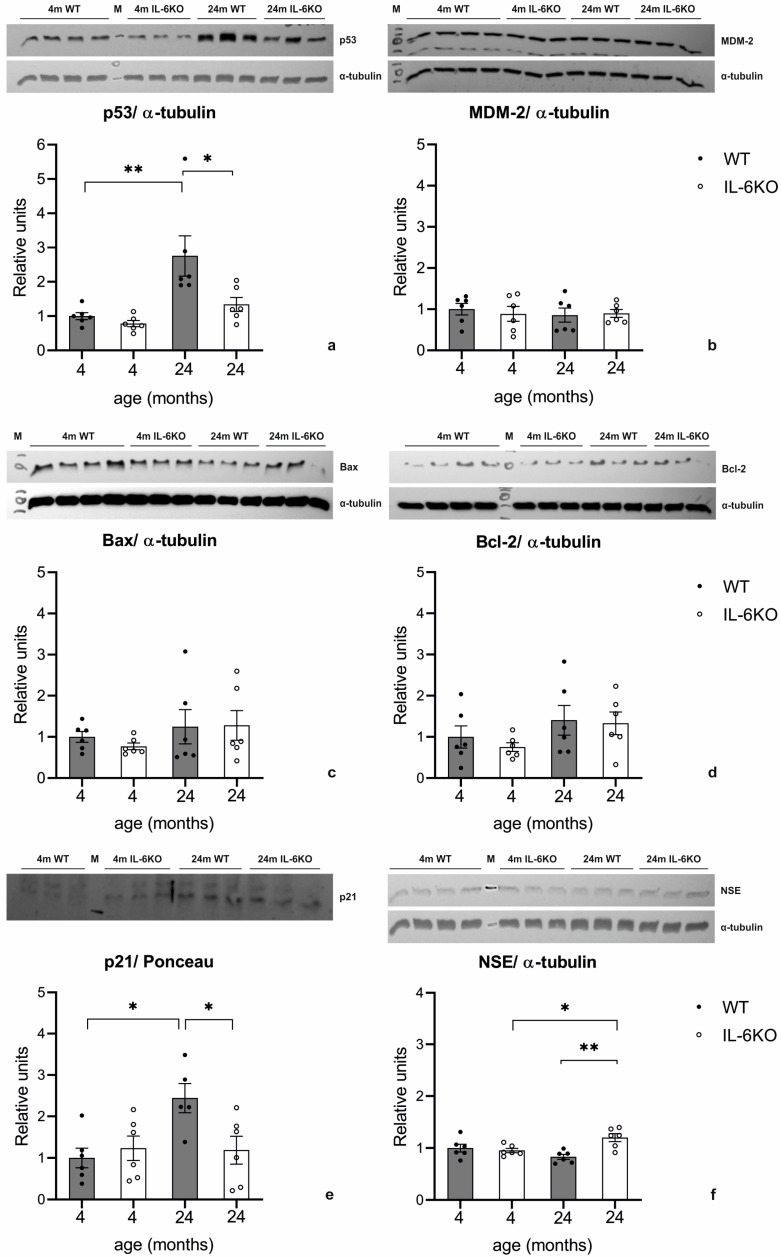
Western blots and densitometric analysis presenting the relative expression of (**a**) p53 protein, (**b**) MDM-2 protein, (**c**) pro-apoptotic Bax protein, (**d**) anti-apoptotic Bcl-2, (**e**) p21 protein, and (**f**) neuron-specific enolase (NSE) protein in cerebellar cortex of 4- and 24-month-old IL-6KO and control WT mice. M—marker. Columns represent the mean ± SEM obtained from 5–6 animals. ANOVA with Tukey post hoc tests revealed that aging significantly increased p53 protein expression in aged WT mice (** *p* < 0.005) but not in IL-6KO mice, compared to genotype-matched younger animals. Aged IL-6KO mice showed lower p53 expression (* *p* < 0.01) than aged WT mice (**a**). No significant changes in MDM-2 expression were observed (**b**). Bax and Bcl-2 levels showed slight, non-significant increases in aged animals (**c**,**d**), respectively). Aging significantly increased p21 protein levels in WT mice (* *p* < 0.05) but not in IL-6KO mice. In aged IL-6KO mice, expression of p21 protein was (* *p* < 0.05) compared to aged WT mice (**e**). NSE levels were comparable across genotypes in young mice. In aged WT mice, NSE expression was significantly lower than in aged IL-6KO mice (** *p* < 0.005), while in aged IL-6KO ones, NSE levels significantly increased in comparison to young adult IL6-deficient mice (* *p* < 0.05) (**e**).

The expression of p53 protein in the cerebellar cortex was similar for the 4-month-old animals of both genotypes. In the 24-month-old animals, p53 expression increased compared to their respective 4-month-old controls in both genotypes. The ANOVA of p53 protein levels yielded *F*_(3,20)_ = 7.802, *p* < 0.005. A Tukey post hoc test revealed a significant increase in p53 expression in the cerebellum of the 24-month-old WT mice compared to both the 24-month-old IL-6-deficient mice (*p* < 0.05) and the genotype-matched younger animals (*p* < 0.005). While p53 levels in the 24-month-old IL-6-deficient mice were higher than those in the 4-month-old IL-6-deficient mice, this difference was not statistically significant (Figure 5a).

Given the observed age- and genotype-related differences in p53 expression determined by Western blotting, the expression of MDM-2, a key regulator of p53, was also analyzed using the same method. MDM-2 expression in the 24-month-old animals remained unchanged compared to the genotype-matched 4-month-old animals, and no significant differences were observed between genotypes (Figure 5b).

The expression of the pro-apoptotic Bax protein in the cerebellar cortex of the 4-month-old IL-6-deficient mice was slightly lower than in the age-matched WT mice, although this difference was not statistically significant. In the 24-month-old animals of both genotypes, a trend toward increased Bax expression relative to the genotype-matched younger animals was observed, but it did not reach statistical significance (Figure 5c).

The expression of the anti-apoptotic protein Bcl-2 was similarly evaluated by Western blotting. In the cerebellar cortex of the 4-month-old IL-6-deficient mice, Bcl-2 levels were marginally lower than those of the age-matched WT mice, though the difference was insignificant. In the 24-month-old animals of both genotypes, a slight increase in Bcl-2 expression was noted compared to the 4-month-old animals, but this change also failed to reach statistical significance. Bcl-2 expression in the aged animals was comparable between genotypes (Figure 5d).

The expression of the p21 protein in the cerebellar cortex was comparable between both genotypes in the 4-month-old animals. However, in the 24-month-old animals, an increase in p21 expression relative to the 4-month-old group was observed exclusively in the WT mice. The ANOVA showed a significant effect on p21 protein levels; *F*_(3,19)_ = 4.260 *p* < 0.02. A Tukey post hoc test revealed a significant increase in p21 protein levels in the cerebellum of the 24-month-old WT mice compared to both the 24-month-old IL-6-deficient mice (*p* < 0.05) and the genotype-matched 4-month-old WT mice (*p* < 0.05). In contrast, the p21 levels in the 24-month-old IL-6-deficient mice remained similar to those in the 4-month-old IL-6-deficient animals (Figure 5e).

NSE expression levels, as determined by Western blotting, were comparable between young adult mice of both genotypes. In the aged animals, NSE expression was significantly lower in the WT mice compared to the IL-6KO mice, though the decrease relative to the 4-month-old WT mice was slight and not statistically significant. The ANOVA analysis of NSE levels yielded F_(3,20)_ = 6.106, *p* < 0.005. Tukey’s post hoc test confirmed a significant decrease in cerebellar NSE protein levels in the 24-month-old WT mice compared to their age-matched IL-6KO controls (*p* < 0.005), as well as a significant increase in NSE levels in the aged IL-6KO mice relative to the younger IL-6KO mice (*p* < 0.05) (Figure 5f).

## 4. Discussion

Our study showed that apoptosis is not a significant factor in the age-related loss of cerebellar cortex cells in 24-month-old mice. Instead, the p53 protein plays a pivotal role in these changes, as evidenced by its significantly higher levels in aged wild-type (WT) mice compared to young WT mice and aged interleukin-6 knockout (IL-6KO) mice. Importantly, aged IL-6KO mice exhibited less severe degeneration of Purkinje cells than aged-matching WT ones, which is likely attributable to reduced neuroinflammation and lower p53 protein abundance. These results suggest that IL-6 deficiency mitigates neurodegenerative processes, potentially protecting against age-related cerebellar damage.

The degree of age-related Purkinje cell loss remains a topic of debate in the literature, with conflicting reports on its extent. However, consistent findings highlight characteristic morphological changes, including constriction of the perikaryon. Studies in rats have shown significant reductions in Purkinje cell volume in 18-, 24-, and 30-month-old animals compared to 3-month-old controls [27]. Similar changes have been reported in aging human brains [28]. The reduction in Purkinje cell volume is thought to stem from a loss of nuclear elements, possibly due to organelle degeneration or cytoplasmic matrix depletion within the perikaryon [29]. In our study, hematoxylin and eosin staining confirmed these findings, showing that Purkinje cells in the 24-month-old animals exhibited shrunken perikarya, eosinophilic cytoplasm, and occasionally pyknotic nuclei, indicative of cellular degeneration. These changes were less severe in the IL-6KO mice, further supporting that IL-6 deficiency plays a protective role. Additionally, autofluorescence of lipofuscin granules—a marker of aging and metabolic stress—was observed in the Purkinje and Bergmann glial cells of the aged mice, suggesting an accumulation of oxidative damage with age. The mitochondria in the 4-month-old mice appeared larger, oval or rod-shaped, and structurally intact. In contrast, the mitochondria in the 24-month-old mice were smaller, polymorphic, and often displayed disrupted cristae or clearings within the mitochondrial matrix. These findings are consistent with previous reports showing a reduction in the number and volume of mitochondria in aging Purkinje cells [30]. Such mitochondrial degeneration may impair the respiratory chain, reducing metabolic activity in these cells [31,32]. These mitochondrial changes also indicate a shift toward autophagic activity. Rather than signifying damage, autophagy likely represents a compensatory mechanism to maintain cellular homeostasis and regenerate damaged components. IL-6 has been shown to influence mitochondrial function in neurons and other cell types, contributing to both neurodegenerative processes and systemic effects. Its role in mitochondrial dysfunction underscores its potential as a therapeutic target for mitigating related pathologies [33,34].

At the ultrastructural level, electron microscopy provided further insights into the cellular changes occurring in aging Purkinje cells. In the young mice of both genotypes, Purkinje cells displayed well-preserved features, including irregular outlines, prominent nucleoli, and well-developed organelles such as mitochondria and the Golgi apparatus. However, in the aged WT mice, significant degeneration was evident. Purkinje cells exhibited irregular nuclear morphology, blurred mitochondrial cristae, and an increased number of lysosomes containing lipofuscin granules. Similar changes were observed in the aged IL-6KO mice, but these alterations were less pronounced, with fewer lysosomes than the WT animals. Other age-related ultrastructural changes were also observed, including the broadening of the cisterns in the smooth endoplasmic reticulum, which serves as the primary intracellular calcium storage site [35,36]. This broadening suggests disturbances in calcium homeostasis, a critical factor in cellular signaling and metabolism. Degeneration of the Golgi apparatus was another prominent feature, accompanied by the accumulation of dense bodies and vacuoles in the cytoplasm. These changes further disrupted cellular homeostasis, leading to the buildup of lipofuscin granules within the cytoplasm and exacerbating the neurodegenerative process [37]. Together, these alterations illustrate how structural and metabolic disruptions in organelles contribute to the progressive degeneration and eventual loss of Purkinje cells in aging brains. Such structural alterations suggest an attenuation of cellular synthesis and a deficit in trophic support, both hallmarks of aging.

Despite the structural deterioration observed in the aging Purkinje cells, apoptosis was not a major contributor to cell loss. Few TUNEL-positive cells were identified in the molecular layer of the aged mice, and active caspase-3 was absent across all cerebellar layers, including Purkinje cells. Expression of pro-apoptotic Bax and anti-apoptotic Bcl-2 proteins showed only minor changes, with no significant shift toward pro-apoptotic activity. These findings suggest that Purkinje cell degeneration results from mechanisms other than apoptosis. Instead, p53 protein accumulation, observed in the aged animals of both genotypes, indicates a role in the DNA damage response (DDR) pathway. Interestingly, the aged WT mice exhibited significantly higher p53 levels than the aged IL-6KO mice, indicating that IL-6 may exacerbate DDR activation. This aligns with the notion that IL-6 deficiency reduces the accumulation of DNA damage, thereby attenuating neurodegenerative processes in aging cerebellar cells. Moreover, the data suggest a relationship where p53 upregulation in the aging WT mice drives the increase in p21 expression, reflecting a typical stress response pathway. In the IL-6-deficient mice, the attenuated p53 response correlates with the lack of significant p21 upregulation, highlighting IL-6’s potential role in facilitating age-related p53–p21 signaling in the cerebellar cortex. This interplay underscores the influence of inflammatory pathways on cellular stress and aging mechanisms.

Compensatory responses to aging were also evident in the cerebellar cortex, particularly in astrocytes. Astrocyte hypertrophy and hyperplasia are well-documented mechanisms activated in response to aging [38,39]. Increased expression of glial fibrillary acidic protein (GFAP), a marker of astrocyte activation, has been observed in various regions of the aging CNS [21,40]. In our study, GFAP immunofluorescence was weak in the astrocytes of the 4-month-old mice but became markedly stronger in the cerebellar cortex of the 24-month-old animals, particularly in the WT mice. This finding was corroborated by Western blot analysis, which showed a significant increase in GFAP protein levels in the aged WT mice compared to the young WT controls. In contrast, although the difference in GFAP levels in the aged animals was insignificant, its increase was less pronounced in the 24-month-old IL-6KO mice than in the younger IL-6KO animals, suggesting that IL-6 deficiency reduces astrocytic activation and associated neuroinflammatory responses.

Insights into the mechanisms of Purkinje cell degeneration come from studies on genetically modified animal models. In the Purkinje cell degeneration (pcd) mice, a spontaneous autosomal recessive mutation in the Nna1 gene leads to toxic substrate accumulation and Purkinje cell loss. This loss was initially thought to involve apoptosis, as TUNEL-positive cells and active caspase-3 were detected [41,42,43]. However, subsequent research suggested that autophagy, rather than apoptosis, plays a dominant role in Purkinje cell death in pcd mice. Elevated levels of LC3-II and reduced levels of LC3-I in the mitochondrial fraction indicated autophagy-mediated neurodegeneration [44]. Similarly, in Lurcher mice, enhanced autophagy, triggered by a mutation in the GluRδ2 gene, was identified as the primary mechanism of Purkinje cell loss [45]. Impaired autophagy has also been implicated in the degeneration of cerebellar cells in Cockayne syndrome, where deletion of the Csb gene results in defective DNA repair, oxidative stress, and an alternative, non-apoptotic cell death pathway [46,47].

Autophagy is a critical cellular response to DNA damage, particularly in neurons. DDR pathway activation, triggered by double-strand breaks (DSBs), is common in aging neurons, including Purkinje cells. Studies have shown that 40–80% of Purkinje cells and 20–40% of cortical and hippocampal neurons in aged mice exhibit DDR features [48]. Key components of this pathway include the p53 protein, which governs a range of stress responses, from DNA repair to apoptosis and cellular senescence [22,23,24,49]. In our study, significant p53 accumulation in the aged mice, particularly the WT animals, indicates DDR activation. Notably, this accumulation occurred without changes in MDM-2 levels, suggesting dysregulation of p53 feedback mechanisms. In the IL-6KO mice, p53 expression was significantly lower, highlighting a potential protective effect of IL-6 deficiency in mitigating DDR activation and DNA damage accumulation.

Under conditions when the cell is not exposed to a stress factor, the p53 protein expression remains at a low level thanks to the MDM-2 protein, which blocks its transcriptional activity by binding to the p53 protein and leads to its degradation by ubiquitination. An imbalance between the level of p53 protein and MDM-2 is a critical point in the activation of p53 protein [50]. p53, when highly expressed or activated due to cellular stress, commonly induces cell cycle arrest and shifts cellular metabolism towards oxidative phosphorylation over glycolysis. NSE is an enzyme in the glycolytic pathway, and increased p53 can shift energy production away from glycolysis, potentially leading to decreased NSE expression as part of glycolysis downregulation. This metabolic reprogramming helps in cellular survival and stress response [25]. In our study, the 24-month-old WT mice showed increased p53 and decreased NSE expression. This likely reflects reduced glycolytic activity and altered energy metabolism, potentially preventing apoptosis in cerebellar cells. The 24-month-old IL-6KO mice exhibit lower p53 expression and higher NSE levels, possibly tied to reduced cellular stress. These changes may attenuate neurodegeneration, suggesting a protective effect due to altered metabolic and stress responses.

Taken together, our results underscore the role of IL-6 in exacerbating age-related cerebellar degeneration and highlight the protective effects of IL-6 deficiency in maintaining cellular integrity during aging. Given that IL-6 deficiency reduces neuroinflammation by inhibiting the STAT3-cGAS-STING pathway in AD models [18] and that IL-6 receptor blockade (e.g., Tocilizumab) prevents neuronal death in PD models [19], targeting IL-6 signaling emerges as a promising neuroprotective strategy. Our findings have broader implications for human neurodegenerative diseases characterized by inflammation-driven neuronal loss such as AD and PD. The observed reduction in neuroinflammation, glial activation, and oxidative stress in IL-6-deficient mice suggests modulating IL-6 signaling could help preserve neuronal integrity and mitigate disease progression.

While our study provides valuable insights into the role of IL-6 in age-related cerebellar changes, some limitations should be considered. Experiments were performed only on male mice at two time points (at 4 and 24 months), and not allowing for full analysis of neurodegenerative changes’ progression. The lack of IL-6 was inborn, potentially allowing for the development of compensatory mechanisms. Finally, while the findings in mice provide essential clues about aging, additional research is needed to determine how well they translate to humans.

## 5. Conclusions

Apoptosis does not significantly contribute to Purkinje cell loss during physiological aging. In the cerebellar cortex of 24-month-old animals from both genotypes, only a few TUNEL-positive cells were detected. This was accompanied by a minor increase in Bax protein expression, no detectable active form of caspase-3, and a significant rise in p53 protein levels. IL-6 deficiency seems to protect against the age-associated neuronal accumulation of pathological changes, which was indicated by less prominent Purkinje cells neurodegeneration and significantly lower p53 protein expression in the cerebellar cortex of the 24-month-old IL-6KO mice compared to the age-matched WT animals. IL-6 deficiency decreases activation of glial tissue in aging, which was indicated by less intensive immunofluorescence of astrocytic GFAP protein in the cells of the cerebellar cortex of the 24-month-old IL-6KO mice than in those of the age-matched WT animals and also supported by its significantly lower protein expression. The results of the present study indicate that inborn IL-6 deficiency slows down the development of age-related pathological changes in the cerebellar cortex triggered by the inflammaging and therapeutic strategies aimed at modulating IL-6 signaling, which could potentially mitigate neurodegeneration and disease progression in neurodegenerative disorders.

## Data Availability

The data of this article will be made available by the corresponding authors on request.

## Abbreviations

AD	Alzheimer’s disease
ANOVA	Analysis of Variance
CNS	Central Nervous System
DDR	DNA Damage Response
GFAP	Glial Fibrillary Acidic Protein
GR	Granular Layer
H+E	Hematoxylin and Eosin
IL-6	Interleukin 6
KO	Knockout
ML	Molecular Layer
NSE	Neuron-Specific Enolase
PC	Polymerase Chain Reaction
PD	Parkinson’s disease
RER	Rough Endoplasmic Reticulum
RT	Room Temperature
SEM	Standard Error of the Mean
TUNEL	Terminal deoxynucleotidyl transferase-mediated dUTP-marker Nick-End Labeling
WT	Wild-Type
